# TFR1 expression in induced sputum is associated with asthma severity

**DOI:** 10.7717/peerj.13474

**Published:** 2022-05-17

**Authors:** Yang Wang, li Feng Gu, Xincheng Zhao, Chengping Hu, Qiong Chen

**Affiliations:** 1 Department of Respiratory Medicine, Xiangya Hospital, Central South University, Changsha, Hunan, China; 2 National Clinical Research Center for Geriatric Disorders, Xiangya Hospital, Central South University, Changsha, Hunan, China; 3 Hunan Children’s Hospital, Changsha, Hunan, China; 4 Xiangya School of Medicine, Central South University, Changsha, Hunan, China

**Keywords:** Asthma, TFR1, M1 macrophages, Asthma severity, Induced sputum, Inflammatory cytokines, Neutrophilic inflammation, Family history, Pulmonary function index, Airway inflammation

## Abstract

**Background:**

Asthma is characterized as a chronic inflammatory airway disease. Iron accumulation is related to asthma pathogenesis. Transferrin receptor 1(TFR1) expression is associated with intracellular iron overload in macrophages. In our study, we explored the association among TFR1 expression, the inflammatory macrophage phenotype, and asthma severity.

**Methods:**

Induced sputum was collected from 50 asthma patients. Real-time PCR was used to evaluate mRNA expression. The status of inflammatory macrophage phenotype was assessed using flow cytometry.

**Results:**

TFR1 levels were inversely correlated with forced expiratory volume in 1 s (FEV_1_)/forced vital capacity (FVC) and FEV_1_/vital capacity (VC). Among inflammatory cytokines, TFR1 expression was positively correlated with IL-1β, TNF-α, IL-6, IFN-γ, and IL-17A mRNA expression in induced sputum. Moreover, TFR1 expression was positively correlated with the number of proinflammatory M1 macrophages and iNOS expression in induced sputum. Neutrophil counts in induced sputum were significantly and positively related to TFR1 expression. Furthermore, TFR1 expression showed an increasing trend in asthma patients with no family history. Our findings indicated that TFR1 expression was consistent with the asthma severity index, especially the proinflammatory M1 macrophage phenotype. TFR1 expression may be a good marker to indicate asthma severity.

## Introduction

Asthma is characterized as a chronic inflammatory airway disease driven by immune cells that leads to symptoms such as wheezing, cough, shortness of breath, and chest tightness (Lambrecht, Hammad & Fahy, 2019). There is a strong correlation between asthma severity and acute asthmatic exacerbation, and most severe asthma patients respond poorly to existing drugs including corticosteroids (CSs) (Bibi et al., 2014; Sullivan et al., 2007). Thus, it is urgent to find an alternative treatment strategy for severe asthma patients. Some reports have indicated that asthma exacerbation is related to an increase in the levels of reactive oxygen-derived species (ROS) and inflammatory cytokines (Henricks & Nijkamp, 2001). In the development of ROS-induced inflammatory conditions, iron serves as the main factor participating in hydroxyl radical production (Carrier et al., 2001). Iron accumulation can result in oxidative stress, lipid peroxidation, and DNA damage (Paul & Lill, 2015; Zhang, 2014). In particular, there is a strong association between dysregulated iron homeostasis and several major respiratory diseases, including asthma (Khadem Ali et al., 2017). Asthmatic lungs were reported to have higher levels of iron, and Zn/Ga−DFO complexes, which are specific iron chelators, had therapeutic effects in a mouse model of asthma (Bibi et al., 2014). In addition, iron overload may facilitate bacterial pathogen invasion and contribute to asthma development (Molyneaux et al., 2014; Starkey et al., 2013b; Starkey et al., 2013a). TFR1 is a type 2 membrane protein expressed in the cell membrane that transfers extracellular iron into the cell (Kuhn, McClelland & Ruddle, 1984; McClelland, Kuhn & Ruddle, 1984; Ghio et al., 2003). A high level of TFR1 expression in the bronchoalveolar lavage fluid (BALF) cells of asthma patients was linked with impaired lung function (Khadem Ali et al., 2020b). However, the specific mechanism by which TFR1 affects asthma exacerbation remains to be clarified.

Iron accumulation was reported to occur mostly in macrophages around the airways in mice with house dust mite (HDM)-induced asthma (Bibi et al., 2014). TFR1^+^ cells were enriched in an asthma mouse model and most TFR1^+^ cells were macrophages (Bibi et al., 2014). These results indicated that macrophages play a crucial role in iron overload-related asthma. There are two types of macrophages that play important roles in asthma: M1 macrophages and M2 macrophages. M1 macrophages can secrete proinflammatory mediators such as tumor necrosis factor (TNF)-α and IL-6 to aggravate lung injury (Kim et al., 2007), while M2 macrophages are beneficial for lung tissue repair and homeostasis in asthma (Moreira et al., 2010). In severe asthma, especially in the steroid-resistant manifestation, M1 macrophages play a leading role in the pathogenesis  (Liu et al., 2014). In summary, targeting M1 macrophages may provide new insights for severe asthma treatment.

To find a novel target for therapeutic treatment for severe asthma , our study for the first time identified whether TFR1 expression was correlated with the asthma severity index, especially the proinflammatory M1 macrophage phenotype in the induced sputum of asthma patients.

## Materials & Methods

### Patients

We recruited 50 asthma patients from Xiangya Hospital, Central South University, China, from June 2021 to November 2021. All asthma patients had a doctor’s diagnosis of asthma and had a history of respiratory symptoms, such as wheezing, chest tightness, cough, expectoration and shortness of breath. All recruited subjects in this study provided written informed consent, and the research protocol was approved by the Medical Ethics Committee of Xiangya Hospital of Central South University (committee reference number: 201803691).

### Acquisition of induced sputum

First, we asked the patients to gargle three times. Then, 4.5% hypertonic saline was used for nebulization. The patients were instructed to expectorate deep sputum between each atomization interval. Sputum mass was collected from the induced sputum. We processed the sputum mass with a four fold volume of 0.1% dithiothreitol by shaking for 30 min at room temperature. We filtered the sputum through 70 µm and 40 µm strainers and centrifuged it at 1,400 rpm for 6 min. The supernatant was discarded, and the sputum cell pellet was resuspended in 2 ml of fluorescence-activated cell sorting buffer (PBS plus 2% fetal bovine serum). The sputum cells were separated into two parts. One part was used for flow cytometry, and the other part was used for RNA extraction.

### Flow cytometry

Sputum cells were blocked with anti-CD16/CD32 antibodies (BioLegend, USA) for 10 min and then stained with Live_Dead (Zomnie Aqua, BioLegend) for 20 min at 4 °C. Human induced sputum cells were then stained with the following antibodies: anti-CD45 (APC/Cy7; BioLegend), anti-CD11c (APC; BioLegend), anti-CD68 (PE; Invitrogen, CN), anti-CD86 (PE/Dazzle594; BioLegend), anti-CD16 (Percp/Cy5.5; BioLegend), anti-CD11b (PE/Cy7; BioLegend), and anti-CD15 (FITC; BioLegend). Flow cytometry was performed with a Cytek Dxp Athena flow cytometer, and the data were analyzed by using FlowJo (version 10) software.

### Quantitative RT–PCR analysis

Total RNA was extracted from sputum cells with trizol reagent (Invitrogen). cDNA was synthesized with HiScript III All-in-One RT SuperMix Perfect for qPCR(Vazyme) according to the manufacturer’s instructions. RT–qPCR was performed with ChamQ Universal SYBR qPCR Master Mix (Vazyme). All primers were purchased from Tsingke Biotechnology (Table 1).

**Table 1:** RT–qPCR primers.

Primers	Sequence (5′–3′)
IL-1β-F	GGGATTCTCTTCAGCCAATCTT
IL-1β-R	ACCACTTGTTGCTCCATATCC
TNF-α-F	GCCTGTAGCCCATGTTGTAG
TNF-α-R	TTGACCTTGGTCTGGTAGGA
TFR1-F	ACCATTGTCATATACCCGGTTCA
TFR1-R	CAATAGCCCAAGTAGCCAATCAT
IL-6-F	CCCTGAGAAAGGAGACATGTAA
IL-6-R	CTCAAATCTGTTCTGGAGGTACT
IFN-γ-F	CTTTGGCTTAATTCTCTCGGAAAC
IFN-γ-R	GTCACTCTCCTCTTTCCAATTCT
IL-17A-F	GGAATCTCCACCGCAATGA
IL-17A-R	TTTGAAGGATGAGGGTTCCTG
iNOS-F	CAAGGTTGTCTGCATGGATAAG
iNOS-R	GGGATCTGAATGTGCTGTTTG
GAPDH-F	GGTCGGAGTCAACGGATTT
GAPDH-R	TCTTGAGGCTGTTGTCATACTT

### Statistical analyses

All results were analyzed with GraphPad Prism 8. The differences between two groups were evaluated using the Mann–Whitney test if the data were nonparametric; otherwise, the unpaired t test was performed. Differences in more than two groups were analyzed with one-way ANOVA followed by Tukey’s HSD post hoc test. When multiple hypothesis testing was performed, Bonferroni adjustment was applied. For correlation analysis, if the data were nonparametric, the Spearman r correlation test was performed; otherwise, Pearson correlation tests were used for the analysis. Differences were considered significant when *p* < 0.05.

## Results

### Patient characteristics

The clinical characteristics of the 50 asthma patients are listed in Table 2. According to the median mRNA expression of TFR1 relative to that of GAPDH, we divided the asthma patients into a TFR1 low expression group and a TFR1 high expression group. There were no significant differences in age (*P* = 0.389), sex (*P* = 0.774), body mass index (BMI) (*P* = 0.831), FEV_1_(%) (*P* = 0.295), FVC(%) (*P* = 0.871), FEV_1_/FVC (*P* = 0.125), FEV_1_/VC (*P* = 0.117), smoking history (*P* = 0.774), asthma control test (ACT) (*P* = 0.382), induced sputum eosinophil cell count (*P* = 0.747), peripheral white blood cell (WBC) count (*P* = 0.563), peripheral neutrophil cell count (*P* = 0.779), or peripheral eosinophil cell count (*P* = 0.344) between the two groups. Obviously, a significantly higher number of neutrophils in induced sputum was observed in TFR1-high subjects than in TFR1-low subjects (*P* = 0.021).

**Table 2:** Clinical characteristics of subjects.

	TFR1 low	TFR1 high	*P* value
**No. of patients (*n*)**	25	25	
**Age (y)**	48.96 ± 11.42	50.24 ± 11.18	0.389
**Sex (male/female), no. (%)**	10(40)/15(60)	11(44)/14(56)	0.774
**BMI**	24.24 ± 3.81	24.17 ± 3.72	0.831
**FEV_1_ (%)**	83.67 ± 19.03	84.92 ± 19.01	0.295
**FVC (%)**	101.55 ± 14.36	102.11 ± 15.13	0.871
**FEV_1_/FVC**	80.82 ± 11.86	81.93 ± 12.37	0.125
**FEV_1_/VC**	85.96 ± 12.35	87.45 ± 12.63	0.117
**Smoking history (never/somker), no. (%)**	14(56)/11(44)	15(60)/10(40)	0.774
**ACT**	16.7 ± 4.03	16.31 ± 3.88	0.382
**Induced sputumn differential count**			
Neutrophils (%)	66.19 ± 21.98	68.46 ± 22.04	0.021
Eosinophils (%)	12.68 ± 14.43	11.57 ± 13.99	0.747
**Peripheral blood**			
WBC (×10^9^/L)	6.63 ± 1.75	6.43 ± 1.79	0.563
Neutrophils (%)	50.35 ± 14.88	50.52 ± 15.01	0.779
Eosinophils (%)	5.07 ± 4.92	4.94 ± 4.93	0.344

**Notes.**

Data are presented as means ± SDs.

BMI Body Mass Index FENO Fractional Exhaled Nitric Oxide ppb parts per billion FEV_1_ Forced Expiratory Volume in 1 s % pred %predicted FVC Forced Vital Capacity VC vital capacity

### Association of the pulmonary function index and TFR1 expression

**Figure 1:**
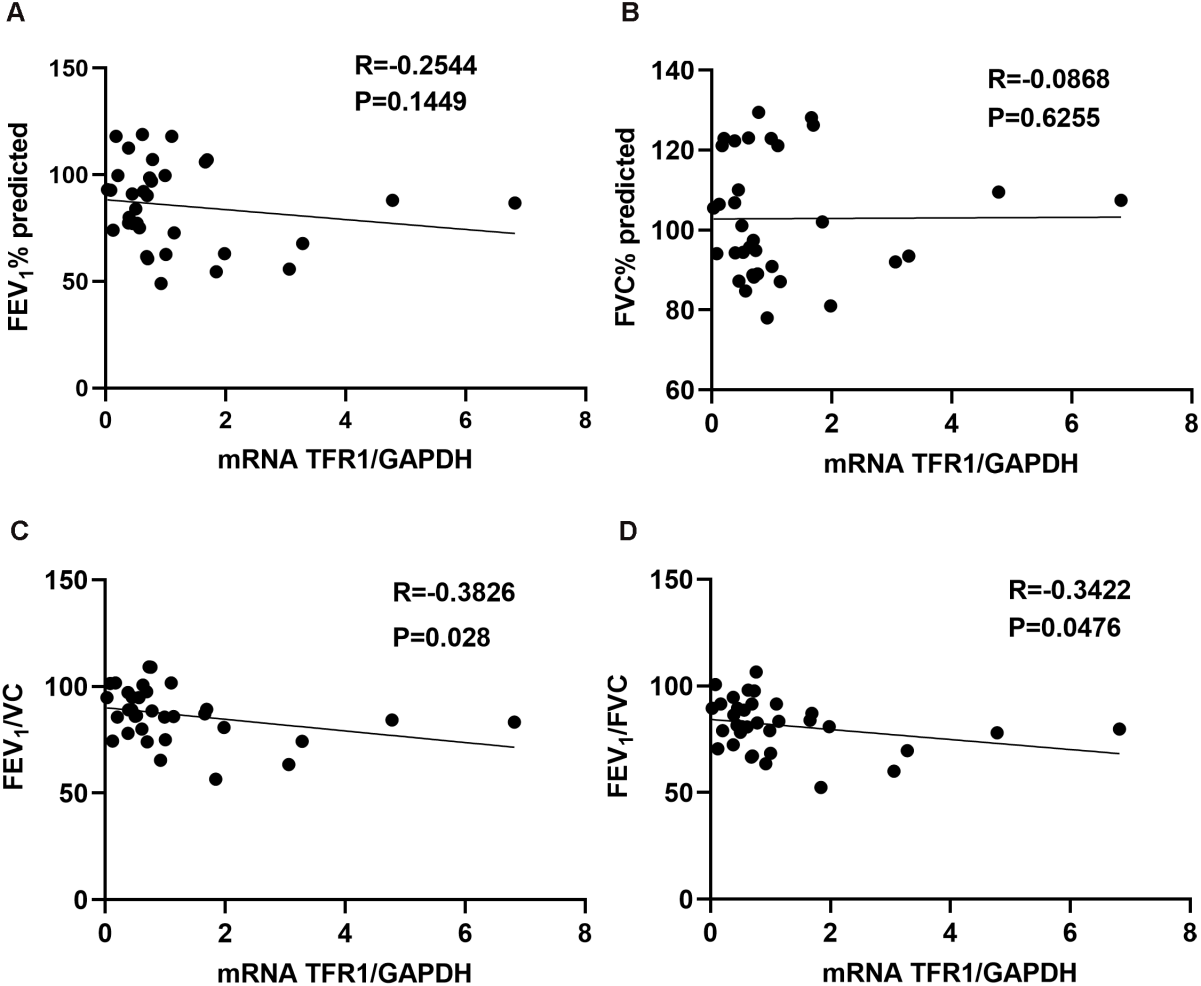
The correlation between TFR1 mRNA expression in induced sputum and the pulmonary function index. (A) Association between TFR1 expression and FEV_1_ (%). (B) Association between TFR1 expression and FVC (%). (C) Association between TFR1 expression and FEV_1_/VC. (D) Association between TFR1 expression and FEV_1_/FVC.

We assessed the relationship between TFR1 expression in induced sputum and the pulmonary function index. TFR1 levels were not correlated with FEV_1_(%) or FVC(%) (Fig. 1A, *R* = −0.2544, *P* = 0.1449; Fig. 1B, R =−0.0868, *P* = 0.6255). However, TFR1 levels were negatively correlated with FEV_1_/VC (Fig. 1C, *R* = −0.3826, *P* = 0.028). Negative relationships were observed between TFR1 levels and FEV_1_/FVC (Fig. 1D, *R* = −0.3422, *P* = 0.0476). Taken together, our results showed that TFR1 levels were associated with declines in lung function in asthma patients, suggesting that TFR1 expression is implicated in asthma severity.

### Relationship of TFR1 expression and inflammatory cytokines

**Figure 2:**
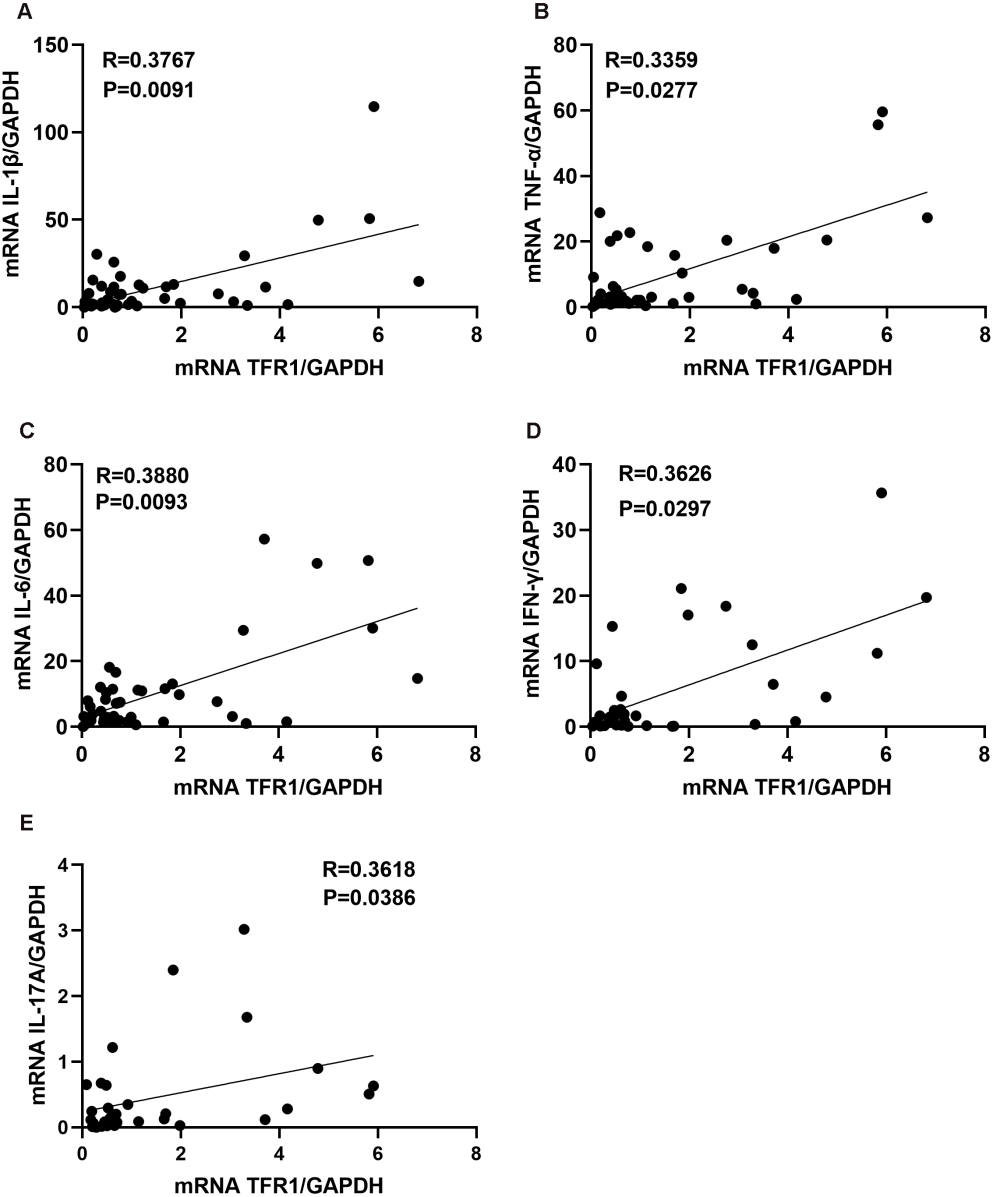
The correlation between TFR1 mRNA expression in induced sputum and inflammatory cytokines. (A) Correlation between TFR1 mRNA expression and IL-1β mRNA expression. (B) Correlation between TFR1 mRNA expression and TNF-α mRNA expression. (C) Correlation between TFR1 mRNA expression and IL-6 mRNA expression. (D) Correlation between TFR1 mRNA expression and IFN-γ mRNA expression. (E) Correlation between TFR1 mRNA expression and IL-17A mRNA expression.

Inflammatory cytokines could promote asthma airway inflammatory cells infiltration, and Th1 inflammatory cytokines (IFN-γ, TNF-α) or Th17 inflammatory cytokines (IL-17A) are more likely to be associated with steroid-resistant asthma (Hansbro, Kaiko & Foster, 2011). We next assessed whether the levels of TFR1 in induced sputum correlated with the inflammatory cytokines. We confirmed that TFR1 expression was moderately and positively correlated with IL-1β, TNF-α, IL-6, IFN-γ, and IL-17A expression in induced sputum (Figs. 2A–2F). The specific correlation R values were 0.3767 (IL-1β, *P* = 0.0091), 0.3359 (TNF-α, *P* = 0.0277), 0.3880 (IL-6, *P* = 0.0093), 0.3626(IFN-γ, *P* = 0.0297) and 0.36183 (IL-17A, *P* = 0.0386) (Figs. 2A–2F). Collectively, these data provide evidence that increased TFR1 expression may play a crucial role in asthma airway inflammation.

### Correlation between TFR1 expression and M1 macrophages

**Figure 3:**
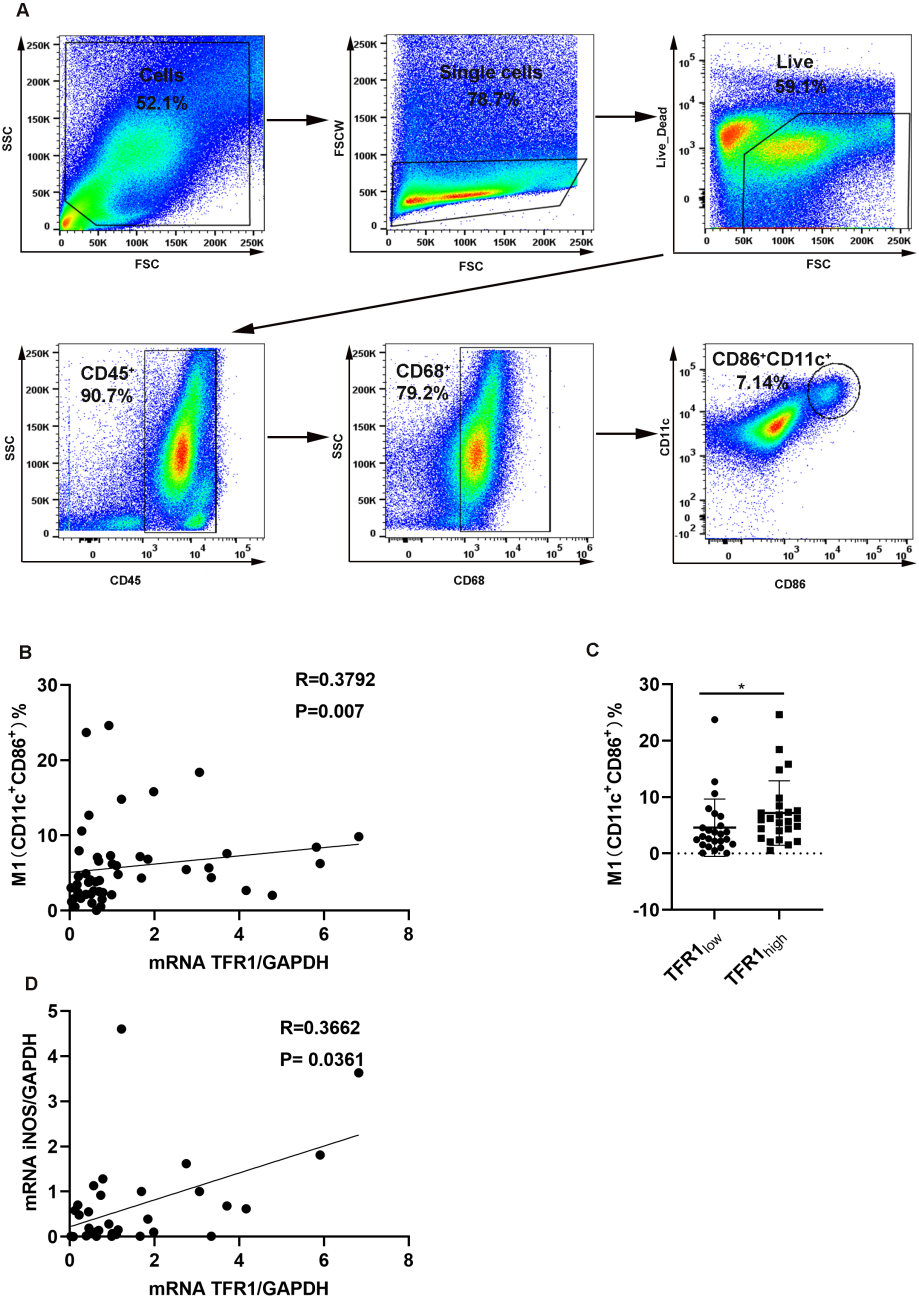
The correlation between TFR1 mRNA expression in induced sputum and M1 macrophage polarization. (A) Representative M1 gating strategy for the induced sputum of asthma patients. (B) Correlation between TFR1 mRNA expression and the number of M1 macrophages. (C) Number of M1 macrophages in the TFR1_high_ and TFR1_low_ groups. (D) Correlation between TFR1 mRNA expression and iNOS mRNA expression. *P < 0.05, **P < 0.01, ***P < 0.001.

It was reported that TFR1 was related to macrophages in an asthma mouse model (Bibi et al., 2014). In addition, proinflammatory mediators including IL-1β, IL-6, and TNF-α, are characteristic features of the M1 macrophage phenotype (Lu et al., 2015), and proinflammatory mediator levels are positively associated with TFR1 expression. We hypothesized that an increase in TFR1 expression might crosstalk with M1 macrophages. First, we analyzed the macrophage population in induced sputum from asthma patients using flow cytometry. Live cells were gated first, and then CD45^+^CD68^+^ cells were gated as macrophages according to previously described methods (Kim et al., 2019). M1 macrophages were then categorized based on the positive expression of CD11c and CD86 among CD45^+^CD68^+^ macrophages (Fig. 3A). We confirmed that TFR1 expression was moderately and positively correlated with the number of M1 macrophages in induced sputum (*R* = 0.3792, *P* = 0.007, Fig. 3B). In particular, the TFR1 high expression group showed more M1 macrophages than the TFR1 low expression group (*P* = 0.0223, Fig. 3C). In addition, iNOS is an M1 macrophage marker. Notably, there was a positive correlation between iNOS expression and TFR1 expression in induced sputum (*R* = 0.3662, *P* = 0.0361, Fig. 3D). Collectively, these novel findings provide evidence that inflammatory M1 macrophage polarization is associated with increased TFR1 expression in asthma patients.

### Association of TFR1 expression with other clinical factors

**Figure 4:**
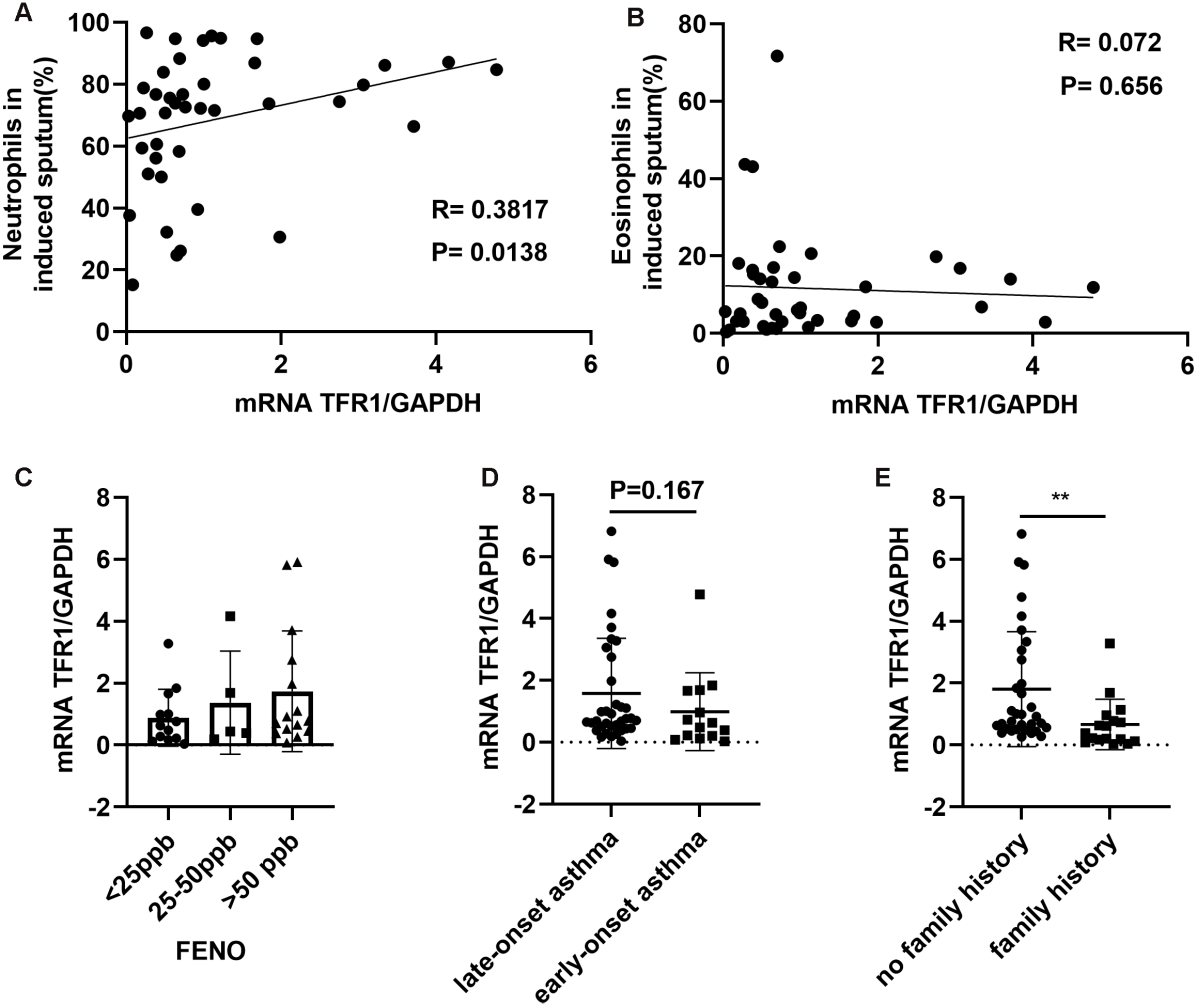
The correlation between TFR1 mRNA expression in induced sputum and other clinical factors. (A) Correlation between TFR1 mRNA expression and neutrophils in induced sputum. (B) Correlation between TFR1 mRNA expression and eosinophils in induced sputum. (C) TFR1 mRNA expression in the three FENO groups. (D) TFR1 mRNA expression in the late-onset asthma group and early-onset asthma group. (E) TFR1 mRNA expression in the no family history asthma group and family history asthma group. *P < 0.05, **P < 0.01, ***P < 0.001.

Among CD45^+^ cells, neutrophils in induced sputum were categorized based on the positive expression of CD15 and CD16, and eosinophils were categorized as CD15^+^CD16^−^, based on previously described methods (Cossarizza et al., 2019). We showed that neutrophil counts in induced sputum were significantly and positively related to TFR1 expression (*R* = 0.3817, *P* = 0.0138, Fig. 4A), while there was no correlation between eosinophil counts and TFR1 expression (*R* = 0.072, *P* = 0.656, Fig. 4B). Fractional exhaled nitric oxide (FENO) was used to identify airway inflammation types according to American Thoracic Society (ATS) clinical practice guidelines (Dweik et al., 2011). Three groups were designated based on FENO: <25 ppb (noneosinophilic airway inflammation), 25-50 ppb (mixed airway inflammation), and >50 ppb (eosinophilic airway inflammation). There was no significant difference in TFR1 expression among the three FENO groups (Fig. 4C). Interestingly, we found that late-onset asthma (LOA) patients showed significantly higher TFR1 expression than other patients, but the difference was not obvious (*P* = 0.167, Fig. 4D). In addition, asthma patients with no family history showed higher TFR1 expression than asthma patients with family history (*P* = 0.0031, Fig. 4E). Taken together, these findings provide evidence that high expression of TFR1 may affect neutrophilic airway inflammation, and the pathogenic effect of TFR1 may occur in patients with no family history.

## Discussion

Upregulated TFR1 expression facilitates iron overload in tissues, which could lead to inflammation progression (Ganz & Nemeth, 2015; Park & Chung, 2019; Marques et al., 2016). TFR1 expression was reported to play a crucial role in disease severity, including asthma pathogenesis (Khadem Ali et al., 2020b). Induced sputum is a safe and noninvasive tool to reflect airway inflammation and the inflammatory phenotype (Szefler et al., 2012). We used induced sputum to examine airway inflammation in our study. In previous studies, the frequency of asthma exacerbation was associated with lung function decline in asthma patients (Bai et al., 2007; O’Byrne et al., 2009), and the loss of lung function reflects airway remodeling (Pascual & Peters, 2005; Kasahara et al., 2002). TFR1 expression in BALF cells was reported to be negatively correlated with FEV_1_/FVC in asthma patients (Khadem Ali et al., 2020b). Similar correlations were observed in our study. Our findings indicated that TFR1 expression in induced sputum had a significantly negative correlation with FEV_1_/FVC and FEV_1_/VC. These results indicate that a higher level of TFR1 expression in induced sputum may be a marker of worse lung function index. However, little is known about how TFR1 plays its important role in the development of asthma airway inflammation.

Inflammatory cytokine production can lead to chronic asthma airway inflammation. IL-6 and IL-1β can amplify airway inflammation and lead to airway epithelial cell dysfunction (Peters & Fahy, 2016; Peters et al., 2016). Studies have indicated that TNF-α levels are increased in asthmatic airways (Bradding et al., 1994; Ying et al., 1991). IFN-γ was reported to be prevalent in severe asthma (Duvall et al., 2017; Raundhal et al., 2015; Oriss et al., 2017). IL-17A is more highly expressed in severe asthma patients than in mild-to-moderate asthma patients and could be a driver of neutrophilic inflammation in the asthmatic airway (Moore et al., 2014; Al-Ramli et al., 2009). Iron overload could promote proinflammatory cytokine secretion to aggravate immune diseases (Wang et al., 2018). Our study was the first to suggest that TFR1 expression has a significantly positive correlation with the levels of Th1/Th17-related inflammatory cytokines (IL-1β, TNF-α, IL-6, IFN-γ, and IL-17A) in induced sputum, indicating that increasing TFR1 expression in induced sputum is related to inflammatory responses in asthma development.

Iron homeostasis in macrophages was reported to be disrupted by inflammation (Sukumaran, Venkatraman & Jacob, 2012; Ganz, 2016). One study showed that higher levels of macrophage infiltration and TFR1 were observed in more ruptured lesions of carotid atheroma, and macrophage levels were significantly related to TFR1 expression (Yuan et al., 2018). Furthermore, pulmonary fibrosis development and lung function decline were related to an increase in TFR1^+^ macrophage counts (Khadem Ali et al., 2020a). M1 macrophages are a kind of macrophages with an inflammatory phenotype that have been reported to have upregulated iron uptake and play a predominant role in severe asthma pathophysiology (Oriss et al., 2017; Tacchini et al., 2008). However, the correlation between M1 macrophage polarization and TFR1 expression remains to be clarified. To identify the mechanism of TFR1 activity in asthma, we first showed that TFR1 expression had a significantly positive association with M1 macrophages in the asthmatic airway. In the TFR1 high expression group, a high number of M1 macrophages were observed. Collectively, our findings are the first suggesting that higher levels of TFR1 expression in the airways are related to higher levels of M1 program macrophages. The correlation between TFR1 and inflammatory M1 macrophages may be a risk factor for severe asthma .

Asthma phenotypes are categorized as neutrophilic, eosinophilic, paucigranulocytic and mixed (Wenzel, 2004). Neutrophilic inflammation in the airway is associated with severe asthma attacks, especially steroid-resistance exacerbation (Moore et al., 2014). It was reported that there was a communication network between Th1/Th17 responses and neutrophilic inflammation in the asthmatic airway (Bullens et al., 2006). The association between the inflammatory factor IL-17 and neutrophils was also observed in the induced sputum of asthma patients (Agache et al., 2010). TNF-α was reported to promote airway inflammation and attract neutrophilic inflammation in the airways (Barnes, 2018). Neutrophils can secrete neutrophil elastase to affect airway remodeling, mucus hypersecretion and lung function decline  (Nadel, 2000; Stănescu et al., 1996). Since our results showed that TFR1 was positively related to TNF-α, IFN-γ, and IL-17A, our study first explored whether TFR1 expression was associated with neutrophil counts in induced sputum. Our findings indicated that neutrophil counts in induced sputum were significantly and positively related to TFR1 expression, while there was no significant association between eosinophil counts and TFR1 expression. Therefore, our study suggests that a high level of TFR1 expression in induced sputum may be a risk factor for neutrophilic asthma.

FENO is strongly recommended for monitoring asthma airway inflammation  (Cowan et al., 2010). One study reported that a low FENO value (<25 ppd) was more likely associated with severe neutrophilic airway inflammation  (Porsbjerg et al., 2009). In the present study, we observed no significant difference in TFR1 expression among the three FENO groups. The underlying reason may be that the assessment of FENO could be influenced by many factors, including smoking status, age, anti-inflammatory drug use, and measurement technique (Borrill et al., 2006). We will investigate this topic more comprehensively in future studies.

The clinical status of patients who develop asthma for the first time in adulthood is known as LOA, but the age definition is not clear (Ayres, 1990). Increasing evidence indicates that LOA patients are more likely to have a high proportion of airflow obstruction (Rossall et al., 2012; Hsu et al., 2004). Another study also showed that LOA patients showed more severe symptoms than early-onset asthma patients (Wu et al., 2015). In our study, the clinical status of the patients who developed asthma at childhood was regarded as early-onset asthma; otherwise, was regarded as LOA. Our results showed that LOA patients had higher expression of TFR1 than early-onset asthma patients, but the difference was not significant. Perhaps we should expand the number of asthma patients to confirm the difference. Parental history, particularly in the case of two parents with asthma, is obviously correlated with early-onset asthma (London et al., 2001). A parental history of asthma is related to early-onset asthma (Panettieri Jr et al., 2008). Interestingly, our findings showed that asthma patients with a family history have lower TFR1 expression than patients with no family history. The underlying reason may be that 66.67% of patients with no family history had nonallergic asthma, while 47% of patients with a family history had nonallergic asthma in our study. Nonallergic asthma has more severe asthma symptoms than allergic asthma (Moore et al., 2010). As for asthma-related exposure in our study setting, we found that four asthma patients with no family history were exposed to a home renovation environment, and two asthma patients with family history were exposed to a home renovation environment. Overall, elevated TFR1 expression may contribute to asthma severity in asthma patients with no family history.

## Conclusions

In conclusion, TFR1 expression is negatively correlated with lung function and is in good agreement with inflammatory cytokine levels. TFR1 expression is associated with proinflammatory M1 macrophage phenotypes and neutrophilic airway inflammation. TFR1 may be a good marker to indicate asthma severity. TFR1 may serve as a potential predictor for asthma severity among asthma patients without a family history.

## Supplemental Information

10.7717/peerj.13474/supp-1 File S1 Dataset

10.7717/peerj.13474/supp-2 Supplemental Information 1 Raw data for Table 2

## Additional Information and Declarations

### Data Availability

The following information was supplied regarding data availability: The raw measurements are available in the Supplementary Files.
